# Trends in reported AIDS defining illnesses (ADIs) among participants in a universal antiretroviral therapy program: an observational study

**DOI:** 10.1186/1742-6405-8-31

**Published:** 2011-09-05

**Authors:** Siavash Jafari, Keith Chan, Kewan Aboulhosn, Benita Yip, Viviane D Lima, Robert S Hogg, Julio Montaner, David M Moore

**Affiliations:** 1 School of Population and Public Health, University of British Columbia, Vancouver, Canada; 2 British Columbia Centre for Excellence in HIV/AIDS, St. Paul's Hospital, Vancouver, Canada; 3 Medical Undergraduate Program, University of British Columbia, Vancouver, Canada; 4 Department of Medicine, Faculty of Medicine, University of British Columbia, Vancouver, Canada; 5 Faculty of Health Sciences, Simon Fraser University, Vancouver, Canada

## Abstract

**Background:**

We examined trends in AIDS-defining illnesses (ADIs) among individuals receiving highly active antiretroviral therapy (HAART) in British Columbia (BC), Canada to determine whether declines in ADIs could be contributing to previously observed improvements in life-expectancy among HAART patients in BC since 1996.

**Methods:**

HAART-naïve individuals aged ≥ 18 years who initiated treatment in BC each of the following time-periods 1996 - 1998; 1999 - 2001; 2002 - 2004; 2005 - 2007 were included. The proportion of participants with reported ADIs were examined for each time period and trends were analyzed using the Cochran-Armitage Trend Test. Cox proportional hazards models were used to examine factors associated with ADIs.

**Results:**

A total of 3721 individuals (81% male) initiated HAART during the study period. A total of 251 reports of ADIs were received from 214 unique patients. These occurred in a median of 4 months (IQR = 1-19 months) from HAART initiation. The proportion of individuals with a reported ADI did not change significantly from 4.6% in the earliest time period to 5.8% in the latest period (p = 0.181 for test of trend). There were no significant declines in any specific ADI over the study period. Multivariable Cox models found that individuals initiating HAART during 2002-04 were at an increased risk of ADIs (AHR = 1.55; 95% CI 1.04-2.32) in comparison to 1996 - 98, but there were no significant differences in other time periods.

**Conclusions:**

Trends in reported ADIs among individuals receiving HAART since 1996 in BC do not appear to parallel improvements in life-expectancy over the same period.

## Background

The introduction of highly active antiretroviral therapy (HAART) in 1996 resulted in significant reductions in HIV/AIDS morbidity and improved survival among HIV-infected individuals compared to the pre-HAART era [1-6]. These improvements in survival were paralleled with reductions in the incidence of AIDS-related opportunistic infections, in the HAART-era compared to earlier time periods [7-9]. This trend is further illustrated by a continued reduction in the proportion of death due to ADI's in HIV infected individuals [10,11].

Life-expectancy of individuals initiating HAART in British Columbia (BC), Canada has continued to increase since the introduction of HAART [1]. In 1996-1998 individuals initiating HAART at the age of 20 years could expect to survive a mean of 11.9 years (standard deviation [SD] = 2.8 years) [1]. By 2002-2004 this life-expectancy at age 20 had increased to 23.6 years (SD = 4.4 years). These findings were confirmed by a large collaboration of ART treatment cohorts examining this same issue [6]. Because prior studies have found that the development of ADIs has resulted in a higher mortality rate among people living with HIV [12,13] one might speculate that the continued increase in the survival of HIV patients in the past 15 years is related to a decrease in the incidence of ADIs. However, to what extent reduced incidence of ADIs during late- HAART eras compared with earlier periods has contributed to this increase in life-expectancy is unknown.

In this study we examined trends in reported ADIs among participants who initiated HAART in the BC HIV/AIDS Drug Treatment Program during the years 1996 to 2007.

## Methods

The BC HIV, Drug Treatment Program (DTP) provides free antiretroviral medications to all medically eligible HIV-infected individuals free-of-charge [1]. Data for this study were drawn from the HAART Observational Medical Evaluation and Research (HOMER) cohort. HOMER is a population-based cohort of antiretroviral-naïve HIV-infected adults 18 years of age and older who are enrolled in the DTP. The current HOMER dataset includes individuals who initiated HAART between August 1, 1996 and February 28, 2009, with follow-up until February 28, 2010. However, we restricted inclusion in this analysis to individuals who initiated HAART before December 31, 2007. Ethical approval for HOMER has been provided by the University of British Columbia Research Ethics Board.

CD4 cell counts were measured using flow cytometry and fluorescent monoclonal antibody analysis (Beckman Coulter, Inc., Mississauga, Ontario, Canada), and HIV viral load was measured using the Roche Amplicor Monitor assay (Roche Diagnostics, Laval, Quebec, Canada). Adherence to HAART was defined as the number of days for which HAART is dispensed divided by the number of days for which HAART is prescribed in the first year of treatment. Deaths are recorded through physician reports and through record linkages between the DTP and the British Columbia Vital Statistic registry. Physicians of DTP participants are mailed a form to assess the clinical stage of HIV disease each year, based on the CDC classification, and are mailed another form if their patient discontinues treatment.

Physician reports of ADIs (CDC Stage C diseases) and patient characteristics were studied for HOMER participants who began treatment in each of the following time-periods: 1996-1998, 1999-2001, 2002-2004, and 2005-2007. We also conducted a data linkage with the provincial cancer registry in order to identify additional AIDS-defining cancers which were not reported by physicians. Patients were followed from the date of starting HAART until the date of ADI (if a condition was reported) or the later of date of death or last laboratory result, to a maximum of 36 months after beginning therapy. The overall reporting rate by physicians was calculated by the number of staging and discontinuation forms returned divided by the number of forms sent.

We compared participant characteristics using Chi-square and Kruskal-Wallis tests. ADI trends and overall reporting trends over time were analyzed using the Cochran-Armitage Trend Test. We calculated 12-months ADI event rates using life tables and constructed Kaplan-Meier curves to examine the time to first ADI diagnosis. Cox proportional hazards models were used to examine independent factors associated with time-to-ADIs. The final multivariate model was constructed using a backward stepwise procedure, with era of HAART initiation forced into the model. To examine the effect of treatment adherence in each era we ran models with and without adherence measures to determine if this affected our results. All analyses were conducted using SAS version 9.1.3 (SAS, Cary, North Carolina, United States).

## Results

A total of 3721 individuals (81% male) initiated HAART during the study period. The median baseline CD4 count was 190 cells/μL (interquartile range [IQR] 90 - 310 cells/μL) and 644 (15%) participants had AIDS at baseline. Table 1 represents the characteristics of participants in our drug treatment program by era of HAART initiation. There were significant differences in the median baseline CD4 cell count (p < 0.001), the gender distribution of participants (< 0.001) and the median age of study participants (p < 0.001) by time-period of HAART initiation but not in the proportion of individuals with a history of injection drug use (p = 0.842).

**Table 1:** Characteristics of participants in the BC HIV/AIDS Drug Treatment Program by era of HAART initiation

ERA (n)	1996-98 (967)	1999-01 (897)	2002-04 (783)	2005-07 (1074)	p-value
**N (%) Male**	829(85.7)	694(77.4)	630(80.5)	863(80.4)	< 0.001

**N (%) with history of ever using injection drugs**	387(40)	341(38)	305(39)	423(39.4)	0.842

**Median age (IQR)**	37 (32-43)	38 (33-45)	42 (35-48)	42 (36-49)	< 0.001

**Median CD4 cell count** **(IQR)**	280 (120-430)	190 (80-330)	150 (70-230)	180 (100-250)	< 0.001

The median follow-up time for all patients was 53 months (IQR 24-101 months) during which there were 251 ADIs reported from 214 patients (Table 2). These occurred in a median of 4 months (IQR = 1-19 months) from HAART initiation. Kaposi's sarcoma (20% of all ADIs), *Pneumocystis jirovecii *pneumonia (17%) and Non-Hodgkin's lymphoma (15%) were the most commonly reported and/or diagnosed ADIs. The proportion of individuals with at least one reported ADI was 4.5% for individuals initiating HAART in 1996-98, 5.7% in 1999 - 2001, 7.8% in 2002-04 and 5.5% in 2005-07. We did not observe a statistically significant trend in the proportion of participants with any ADI over the study period (p = 0.130, for test of trend), or any cause-specific ADI. The median CD4 cell count for those with ADIs were 140; 95; 70 and 80 cells/μL for each time period (p = 0.213). The 12-month probability of reported ADIs in each time-period was as follows: 1996-98 = 0.020 (95% confidence interval [CI] 0.011-0.029); 1999-2001 = 0.038 (0.025-0.050); 2002-04 = 0.059 (0.042-0.076); 2005-07 = 0.047 (0.034-0.060). A similar trend was also reflected in the Kaplan-Meier analysis of ADI-free survival which found significant differences between the different periods of HAART (log-rank test p = 0.008), with the 2002 - 2004 period having the highest risk of ADI (data not shown).

**Table 2:** Summary of reported AIDS-defining illnesses (ADI) by year of HAART initiation

ERA (n)	1996-98 (967)	1999-01 (897)	2002-04 (783)	2005-07 (1074)	Total (3721)	p-value
**Any ADI (%)**	44 (4.5)	50 (5.6)	61 (7.8)	59 (5.5)	214 (5.75)	0.181

**Kaposi's Sarcoma (%)**	7 (0.7)	10 (1.1)	13 (1.7)	12 (1.1)	42 (1.1)	0.295

**Pnemocystis jirovecii pneumonia (%)**	12 (1.2)	8 (0.9)	7 (0.9)	9 (0.8)	36 (0.97)	0.386

**Non-Hodgkin's Lymphoma (%)**	9 (0.9)	9 (1.0)	6 (0.8)	8 (0.7)	32 (0.86)	0.552

**Mycobacterium avium intracellae (%)**	6 (0.6)	6 (0.7)	11 (1.4)	5 (0.5)	28 (0.75)	0.971

**HIV Wasting Snydrome (%)**	3 (0.3)	3 (0.3)	6 (0.8)	8 (0.7)	20 (0.54)	0.103

**Mycobacterium Tuberculosis (%)**	0 (0)	7 (0.8)	4 (0.5)	4 (0.4)	15 (0.40)	0.363

**Cryptococcal Meningitis (%)**	3 (0.3)	2 (0.2)	5 (0.6)	3 (0.3)	13 (0.35)	0.785

n = number who initiated HAART in each period

In the multivariable model (Table 3), we found that individuals who initiated HAART in 2002-04 were at an increased risk for ADIs (adjusted hazard ratio [AHR] = 1.55; 95% CI 0.81-1.88) in comparison to 1996-98; There were no significant associations with the time-periods of 1999-2001 (AHR = 1.24 (95% CI 0.81-1.88) or 2005 - 07 (AHR = 1.26 (95% CI 0.85-1.88) in comparison to 1996-98. Factors which were associated with risk for ADI included baseline CD4 counts < 50 cells/μL, (AHR = 3.48; 95% CI 2.43-4.99) and between 50 - 199 cells/μL (AHR = 1.60; 95% CI 1.13-2.26), baseline HIV viral load (AHR = 2.03 per log_10 _increase; 95% CI 1.47-2.79); and the inclusion of NNRTIs in the first drug regimen (AHR = 0.77; 95% CI 0.56-1.05). A multivariate model which included adherence to therapy also found no difference in risk for ADIs associated with the time-period in which participants initiated HAART.

**Table 3:** Cox proportional hazards analysis of time to first AIDS event following initiation of HAART by period of HAART initiation

Variable	Unadjusted Hazard Ratio (95% CI)	p-value	Adjusted Hazard Ratio (95% CI)	p-value	Adjusted Hazard Ratio (95% CI)	p-value
			**w/adherence**		**w/o adherence**	

Age (per decade)	1.16 (1.01-1.32)	0.031	1.18 (1.03-1.35)	0.020		

Gender		0.133				
Female	1.00					
Male	1.33 (0.92-1.94)					

Baseline AIDS defining illness	1.93 (1.42-2.63)	< 0.001				

CD4 (per 100 cells)	0.64 (0.57-0.72)	< 0.001				

Baseline CD4						

< 50	4.76 (3.38-6.70)	< 0.001	3.56 (2.48-5.11)	< 0.001	3.48 (2.43-4.99)	< 0.001
50-199	1.93 (1.37-2.70)	< 0.001	1.61 (1.14-2.28)		1.60 (1.13-2.26)	0.008
200+	1.00		1.00	0.007	1.00	

Baseline Viral Load (log10)	3.32 (1.99-5.54)	< 0.001	2.07 (1.50-2.85)	< 0.001	2.03 (1.47-2.79)	< 0.001

Baseline Viral Load		< 0.001				
< 100,000	1.00					
> 100,000	2.67 (1.96-3.64)					

Third drug of baseline therapy		0.003		0.073		0.096
PI	1.00		1.00		1.00	
NNRTI	0.64 (0.47-0.86)		0.75 (0.55-1.03)		0.77 (0.56-1.05)	

Hepatitis C		0.595				
Negative	1.00	0.560				
Positive	1.08 (0.81-1.43)					
unknown	1.15 (0.73-1.81)					

History of Injection drug use	1.12 (0.85-1.46)	0.424				

Year therapy started (per year increase)	1.03 (0.99-1.07)	0.189				

ERA therapy started						
96-98	1.00		1.00		1.00	
99-01	1.25 (0.83-1.87)	0.286	1.25 (0.82-1.91)	0.293	1.24 (0.81-1.88)	0.320
02-04	1.77 (1.20-2.60)	0.004	1.59 (1.06-2.39)	0.025	1.55 (1.04-2.32)	0.032
05-07	1.26 (0.85-1.86)	0.251	1.36 (0.90-2.04)	0.141	1.26 (0.85-1.88)	0.255

One-year Adherence (per 10% increase)	0.89 (0.86-0.93)	< 0.001				

One-year Adherence		< 0.001		< 0.001		
< 95%	1.00		1.00			
≥ 95%	0.50 (0.38-0.65)		0.42 (0.32-0.56)			

We reviewed the number of physician's reports submitted for other programmatic reasons such as clinical staging or medication discontinuation forms to see if ADI reports could have been influenced by changes in overall physician reporting. Our results indicate that the number of other physician reports decreased significantly over the study period with 63% of physicians submitting at least one report in 96-98, 62% in 1999-2001, 44% in 2002-04 and 49% in 2005-07.(p-value < 0.001).

## Discussion

The proportion of individuals with reported ADIs within 36 months of treatment initiation has not changed significantly among individuals accessing HAART in BC over a 12-year period. Considering that the baseline CD4 remained relatively constant, it was not surprising that the incidence of reported ADI's did not significantly change. However, this result is somewhat unexpected given the improvements in life-expectancy we have seen in the same period in this population [1]. Additionally our observed bias toward decreased overall reporting in recent years from physicians in our program further supports our conclusion that ADI rates have not decreased over this period. Therefore, it appears that improvement in life expectancy of HIV/AIDS patients in this period is due to factors other than a decrease in the incidence of ADIs. Most likely this is due to reductions in non-AIDS related conditions, but may also be related to other factors, as well.

The importance of reductions in non-AIDS related conditions contributing to improvements in clinical outcomes has been previously highlighted by the SMART [14,15] study which found that continuous treatment with HAART decreases the risk of major cardiovascular, renal and hepatic diseases and mortality rate among people with HIV.

We did find that individuals who initiated treatment during 2002-04, did have an increased risk of being diagnosed with an ADI. However this does not appear to be part of a trend towards an increased or decreased risk over time. It is noteworthy that this time-period was characterized by the lowest median baseline CD4 cell counts (150 cells/μL), but that this relationship persisted even after adjustment for baseline CD4 counts. Since this period was prior to the release of the SMART data, when medically supervised treatment interrupted were considered a reasonable part of clinical management, it is possible that such interruptions may have contributed to the increase incidence of ADIs during this period.

Our results contrast somewhat with a recent examination of rates of ADIs among participants in the HIV Outpatient Study, which did find significant reductions in the incidence of ADIs between 2003-2007 in comparison to 1998-2002 [16]. The HOPS Study had a larger number of participants (approximately 9000 in the period after 1998), but did not restrict their study to individuals who initiated HAART in each time period, therefore the two studies are not directly comparable. Our study was specifically designed to look at the effects of the changing management and medications associated with initiating HAART in each time period, rather than overall ADI incidence rates.

While we did not find significant changes in the rates of ADIs in later time-periods, a significant minority of HAART patients continue to experience serious illness even in the latest time period. Early linkage of HIV patients to care and better adherence to treatment plan have been shown to prevent the development of ADIs [17] and improve clinical outcome [18]. Fortunately, the median CD4 count at initiation increased in the last time period and more recent analyses have shown that this has now climbed to above 200 cells/μL [19]. These findings highlight the need for better strategies to facilitate earlier identification of HIV-infected individuals and link them to care in BC. Such strategies would likely result in even further improvements in life-expectancy for HIV-infected individuals.

There are several limitations to our study. Firstly, the number of ADIs reported in each time-period was quite small which limited our ability to detect significant changes in reported cases. Secondly, we expect that physicians underreport ADIs events, however, this underreporting appears to be greatest in the later time-periods which should have biased our results towards showing significant declines, and instead the ADI rate remained statistically unchanged. Conversely, it is also possible that physicians have become more astute or vigilant about reporting AIDS over time. Third, there is a possibility of variations in the quality of reports of cases with the ADI. This can be less of a problem for ADIs with clear diagnostic criteria (TB, cryptococcal disease or cancers) than with more subjective diagnoses (wasting). Lastly, as with all observational studies the lack of difference we have observed may be confounded by other factors which differ between the time-periods and we are unable to measure.

## Conclusions

The overall incidence of ADIs after HAART has not changed significantly after the introduction of the HAART in BC. These observations suggest that previously described recent improvements in the life expectancy among patients initiating HAART might have been because of reductions in the occurrence of other non-AIDS related clinical conditions. Further research is needed to examine this hypothesis.

## Competing interests

JSGM has received funding from Merck, Gilead and ViiV Healthcare to support research into Treatment as Prevention, consultancy fees from Merck, and speakers' fees from Clinical Care Options. RSH has received a research grant from Merck and a conference travel grant from GlaxoSmithKline. None of the other authors have any known competing interests.

## Authors' contributions

DMM, JSGM, RSH and SJ conceived of the study, and participated in its design and coordination. BY, VL and RSH supervised the data collection and the preparation of the dataset for analysis. KC conducted all of the data analysis. SJ wrote the first drafts of the paper, incorporated the comments of the other authors and was assisted by KA. All authors approved the final version of the manuscript for submission.
